# Vanishing Bile Duct Syndrome in Hodgkin's Disease: case report

**DOI:** 10.1590/S1516-31802000000500008

**Published:** 2000-09-01

**Authors:** Marta Soares Rossini, Irene Lorand-Metze, Gislaine Borba Oliveira, Cármino Antonio De Souza

**Keywords:** Hodgkin's disease, Vanishing bile duct syndrome, Liver failure, Doença de Hodgkin, Síndrome do desaparecimento dos dutos biliares, Falência hepática

## Abstract

**CONTEXT:**

Liver damage is relatively common in patients affected by Hodgkin's disease. A smaller proportion of cases develops jaundice. Recently, the vanishing bile duct syndrome was described in Hodgkin's disease. The mechanisms of this severe complication have been poorly understood until now.

**OBJECTIVE:**

To describe a rare case of intra-hepatic cholestasis due to vanishing bile duct syndrome.

**DESIGN:**

Case report.

**CASE REPORT:**

A 38-year-old male patient affected by Hodgkin's disease. Liver biopsy showed no detectable Hodgkin's disease. Intra-hepatic cholestasis was found and none of the six portal tracts analyzed contained normal bile ducts. The treatment was based on conventional and high-dose escalation chemotherapy. The patient died from an irreversible liver failure while in complete remission from Hodgkin's disease.

## RESUMO

**CONTEXTO:**

Lesões no fígado são relativamente comuns em pacientes com a Doença de Hodgkin. Uma pequena proporção dos casos desenvolvem icterícia. Recentemente, a Síndrome do desaparecimento do duto biliar desenvolveu-se na Doença de Hodgkin. Os mecanismos dessa doença são muito pouco conhecidos até hoje.

**OBJETIVO:**

Descrever um raro caso de colestase relacionada à síndrome do desaparecimento dos dutos biliares.

**TIPO DE ESTUDO:**

Relato de Caso.

**RELATO DO CASO:**

Paciente masculino, 38 anos, portador de Doença de Hodgkin. A biópsia hepática revelou ausência de HD e colestase intra-hepática sendo que dos seis espaços porta analisados nenhum apresentava dutos biliares normais. O tratamento foi baseado em quimioterapia convencional e posterior quimioterapia em altas doses escalonadas. O paciente apresentou uma má evolução e faleceu devido a falência irreversível da função hepática, em remissão completa do linfoma.

## INTRODUCTION

Liver damage is relatively common in patients affected by Hodgkin's disease. Non-specific inflammation in portal areas is seen in approximately 50% of liver biopsies, but Reed-Sternberg cells can be demonstrated in about 5% of them.^1^ However, a smaller proportion of cases develop jaundice.^2^ Other causes of cholestasis in Hodgkin's disease include biliary obstruction (by lymph node enlargement), hemolysis, viral hepatitis and drug toxicity. In the past, many cases that presented no detectable cause of jaundice were called “idiopathic jaundice”. Recently, the vanishing bile duct syndrome^2,3^ was described in Hodgkin's disease. The mechanisms of this severe complication have been poorly understood until now.^2^ However, this syndrome has been described in many other diseases such as primary biliary cirrhosis, graft-versus-host-disease or primary sclerosing cholangitis.^4,5^ Vanishing bile duct syndrome is also a major histological finding in rejection after liver transplantation.^2,4^ Advanced vanishing bile duct syndrome is probably a manifestation of irreversible liver damage.^2–6^ We describe a case of Hodgkin's disease associated with vanishing bile duct syndrome.

## CASE REPORT

**Figure 1:**
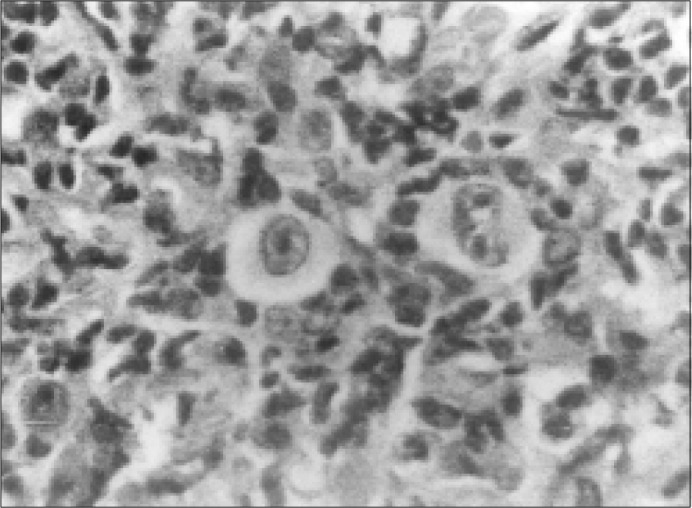
Lymph node biopsy specimen from patient showing typical Hodgkin's Disease, mixed cellularity sub type.

**Figure 2:**
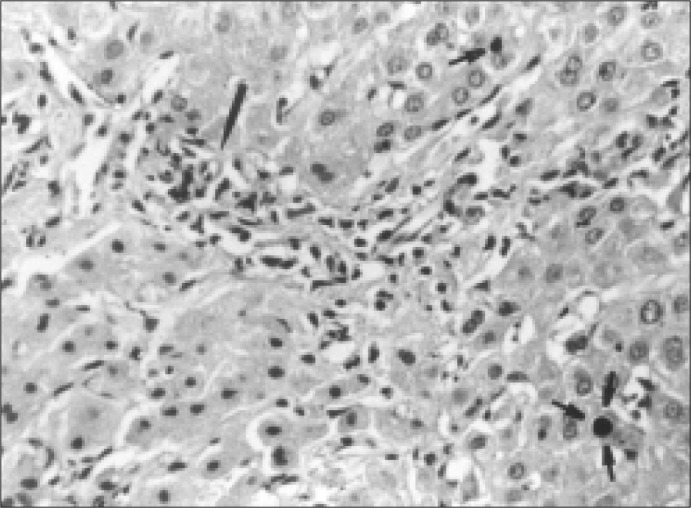
Liver biopsy specimen from the patient. Portal space showing slight mononuclear inflammatory infiltrate. Long arrow shows arteriole without intra-lobular duct. Short arrows show biliary parenchyma plug.

A 38-year old male was seen at the General Hospital of the State University of Campinas, Brazil, in December 1997. He presented a four-week history of cervical lymphadenopathy, jaundice and pruritus. The lymph node histology showed typical Hodgkin's disease, with mixed cellularity (Figure 1). The abdominal computed tomography revealed only hepatomegaly. The X-ray of the thorax and bilateral bone marrow biopsies were normal. Hb = 15.6 g/dl; WBC = 7.6 x 10^9^/L; Platelets 392 x 10 ^9^/L. At the time of admission the biochemical values indicated cholestasis without signs of hepatic failure (Table) Serological tests for hepatitis A, B and C, HIV and cytomegalovirus were negative. Needle biopsy of the liver (Figure 2) showed intra-hepatic cholestasis. Interlobular bile ducts were absent in all of the six portal tracts examined. Involvement by Hodgkin's disease could not be detected. The patient was treated with standard chemotherapy (four cycles of MOPPABV^7^). Cholestasis progressively increased with signs of hepatic failure (decrease of serum albumin level and elongation of the prothrombin time). During chemotherapy, the patient presented enlargement of a left axillary lymph node. Then, salvage therapy using high dose Cyclophosphamide 7 g/m² (HD-CY) followed by Etoposide 2 g/m² (VP-16-213) was given to reduce tumor burden and to collect peripheral blood progenitor cell in order to perform autologous bone marrow transplantation. The lymphadenopathy disappeared and there was a short period of stabilization of hepatic function and cholestasis. One month later there was deterioration of the liver function and the patient presented an episode of massive hematemesis with dehydration and acute renal failure. Although a liver transplantation was considered, the patient died due to hepatic failure.

**Table:** Biochemical changes and chemotherapy in the Hodgkin's disease patient

	Dec 97	Feb 98	Mar 98	Apr 98	May 98	Jun 98	Jul 98	Normal
Total bilirubin (μmol/l)	33.5	29.6	37.1	31.5	32	25.2	46	<1.2
Direct bilirubin (μmol/l)	22.7	20.6	24.5	20.5	22	16.3	24	<0.6
AST (U/l)	203	221	194	252	225	45	210	6-18
ALT (U/l)	203	319	170	219	130	41	84	4-22
Alkaline Phosphatase (U/l)	2041	1494	3566	5613	5804	2964	7970	65-200
γ-GT (U/l)	809	1167	1972	2539	2085	1039	1860	4-23
Albumin (g/dl)	3.67	-	-	3.78	3.5	3.8	2.7	3.5-5.5
PT (INR)	1.31	1.31	1.42	-	2.8	1.0	2.24	1.0
R			0.83			1.33	5.41	1.0
Fibrinogen	300							160-450
Chemotherapy	MOPP/ABV	MOPP/ABV	MOPP/ABV	MOPP/ABV	HDCY	VP-16	Death	-

## DISCUSSION

Liver involvement is uncommon in Hodgkin's disease at diagnosis. Cervantes et al^8^ found 7.4% of liver involvement in Hodgkin's disease in 421 cases studied. Infiltration is defined by the presence of Reed Sternberg cells usually accompanied by lymphocytes, histiocytes, eosinophils and plasma cells in the portal tracts. This infiltration of the liver can lead to cholestasis and jaundice. Other causes of jaundice in Hodgkin's disease include extra-hepatic biliary obstruction by enlarged portal lymph nodes, hemolysis, viral hepatitis and drug toxicity.^4^ Recently, the vanishing bile duct syndrome has been described as a rare and severe cause of intra-hepatic cholestasis in Hodgkin's disease.^2,3^

This syndrome consists of the destruction of the biliary apparatus with the disappearance of the small and medium-sized intra-hepatic bile ducts.^2,3,5^ It has been observed in different congenital or acquired diseases such as chronic rejection of liver transplantation, resulting in graft failure in 5% to 20% of allograft recipients; graft-versus-host-disease after bone marrow transplantation; primary biliary cirrhosis; primary sclerosing cholangitis; chronic drug-induced cholestasis (clindamycin; carbamazepine; trimethoprim-sulphamethoxazole) and histiocytosis X in children.^4-6,9-11^

Hubscher, et al.^2^ described three cases of Hodgkin's disease that presented reduction of biliary ducts. The three cases died with intractable liver damage. The first case, a 26-year-old man (Hodgkin's disease of the nodular sclerosing type), presented a two-week history of jaundice, fatigue and weight loss. Laboratory studies showed severe cholestasis, although abdominal ultrasound examination showed no evidence of biliary obstruction. He was treated with chemotherapy. He died 24 weeks later with hepatic encephalopathy, renal failure, severe diarrhea and neutropenia. The cause of the death was disseminated fungal infection. No residual lymphoma was detected in any of the organs examined at autopsy. The second case, a 44-year-old man, presented a 2-week history of jaundice. The liver biopsy showed intrahepatic cholestasis of unknown pathogenesis. The diagnosis of Hodgkin's disease (lymphocyte predominant type) was made 7 months later. At this time the jaundice got worse and the liver biopsy showed involvement by Hodgkin's disease. He began treatment with radiotherapy and chemotherapy and died three days later. The third case, a 37-year-old woman presented pruritus, weight loss, persistent cough and night sweats. The diagnosis of Hodgkin's disease of the nodular sclerosing type was made by thoracotomy. Ten days after chemotherapy she developed jaundice. The ultrasound examination was normal. Liver function deteriorated and the patient developed renal and respiratory failure and died. At autopsy no residual lymphoma was detected. In the three cases cholestasis and paucity of bile ducts were noted in the liver biopsies. Only case 2 showed evidence of lymphomatous infiltration. Gottrand et al described a 3.5-year-old child with a three-week history of submaxillary lymphadenopathy. The lymph node biopsy showed a mixed cellularity Hodgkin's disease. On admission she had jaundice without hepatosplenomegaly. The biochemical values were consistent with cholestasis. No infiltration by Hodgkin's disease was found at the liver biopsy. There was a paucity of interlobular bile ducts. Cholestasis progressively increased without any signs of hepatic failure. Chemotherapy and radiotherapy were given. Five months later, the lymphadenopathy had regressed, but cholestasis continued to increase. Liver transplantation was considered, but the patient died with signs of hepatic failure.

In all these four cases described in the literature, as well as in the present one, vanishing bile duct syndrome was detected at diagnosis or at least when Hodgkin's disease showed tumor activity. However, even after a good response to chemo-therapy, and complete remission of Hodgkin's disease, vanishing bile duct syndrome progressed. All patients died from irreversible hepatic failure. At autopsy, no evidence of Hodgkin's disease was found.

The pathophysiology of vanishing bile duct syndrome is not well understood. Immunological mechanisms seem to be involved. Hubscher, et al.^2^ suggested that there is a release of toxic cytokines from lymphoma cells in Hodgkin's disease. The destruction of bile ducts in primary biliary cirrhosis, primary sclerosing cholangitis and liver allograft rejection seems to be related to cell-mediated immunological attack by cytotoxic T lymphocytes of either CD4 or CD8 phenotype. Other investigations have indicated the presence of immunoglobulins in interlobular bile ducts, suggesting the involvement of humoral immune reactions. Hodgkin's disease may be associated with autoimmune manifestations but, even with complete remission of Hodgkin's disease after chemotherapy, vanishing bile duct syndrome is irreversible, probably due to the fact that the affected bile ducts have a low regeneration capacity.

Patients with liver disease as the initial manifestation of Hodgkin's disease have a poor prognosis. In particular, vanishing bile duct syndrome is a progressive and always fatal complication in this setting, although some reversible cases have been described in association with other liver transplantations.^12^ Liver transplantation for vanishing bile duct syndrome in Hodgkin's disease should be considered. Our patient died before any procedure could be done.
